# Insight into the Genetic Population Structure of Wild Red Foxes in Poland Reveals Low Risk of Genetic Introgression from Escaped Farm Red Foxes

**DOI:** 10.3390/genes12050637

**Published:** 2021-04-25

**Authors:** Heliodor Wierzbicki, Magdalena Zatoń-Dobrowolska, Anna Mucha, Magdalena Moska

**Affiliations:** Department of Genetics, Wrocław University of Environmental and Life Sciences, 51-631 Wrocław, Poland; magdalena.zaton-dobrowolska@upwr.edu.pl (M.Z.-D.); anna.mucha@upwr.edu.pl (A.M.); magdalena.moska@upwr.edu.pl (M.M.)

**Keywords:** admixture, genetic introgression, microsatellites, mtDNA, red fox

## Abstract

In this study we assessed the level of genetic introgression between red foxes bred on fur farms in Poland and the native wild population. We also evaluated the impact of a geographic barrier and isolation by distance on gene flow between two isolated subpopulations of the native red fox and their genetic differentiation. Nuclear and mitochondrial DNA was collected from a total of 308 individuals (200 farm and 108 wild red foxes) to study non-native allele flow from farm into wild red fox populations. Genetic structure analyses performed using 24 autosomal microsatellites showed two genetic clusters as being the most probable number of distinct populations. No strong admixture signals between farm and wild red foxes were detected, and significant genetic differentiation was identified between the two groups. This was also apparent from the mtDNA analysis. None of the concatenated haplotypes detected in farm foxes was found in wild animals. The consequence of this was that the haplotype network displayed two genetically distinct groups: farm foxes were completely separated from native ones. Neither the River Vistula nor isolation by distance had a significant impact on gene flow between the separated wild red fox subpopulations. The results of our research indicate a low probability of genetic introgression between farm and native red foxes, and no threat to the genetic integrity of this species.

## 1. Introduction

Many wild animal populations are endangered by genetic introgression from domesticated animals that have escaped from farms [1,2,3,4,5,6,7,8]. Genetic introgression can be defined as the incorporation of alleles from one species into the gene pool of a second, divergent species [9]. This can lead to outbreeding depression significantly disrupting locally adapted gene complexes and to extinction by genetic replacement [10,11,12]. As a result of the human-mediated movement of animals, it is becoming increasingly important to understand and manage genetic introgression and its effects [13]. Accidentally or intentionally introduced wild or domestic species can interbreed with their domesticated counterparts [3,14,15,16]. Gene flow from domesticated animals may carry alleles that may increase the fitness of the wild animals, so natural selection will drive introgression [3]. However, a long-term concern is that introgression may lead to a loss of genetic integrity in native species whose ranges are invaded by non-native individuals. An increasing conservation problem is the admixture of native populations with newcomers (e.g., animals bred by humans on farms scattered across the country). In this situation, introgression is not restricted to the distributional margins, but is catalysed by spots of hybridization inside the range of a native species [17].

The distributions of species are altered and disturbed by humans in many ways, ranging from transfer of animals between continents to conversion and destruction of habitats. The introduction of non-native species from intercontinental translocations is particularly harmful to native populations and is considered one of the main causes of biodiversity decline [18,19].

The red fox (*Vulpes vulpes*) is currently not an endangered species (its conservation status is Least Concern, [20]). However, it could lose its genetic integrity through introgressive hybridization, a threat that comes from non-native red foxes originating primarily from fur farms. Farm-reared red foxes can penetrate local populations, either directly as escapees or indirectly, where individuals released as hunting stock are sourced from captive-reared, fur farms [21,22,23].

The wild population of the red fox in Poland, estimated at 200,000–250,000 individuals [24], is particularly vulnerable to introgressive hybridization because this country is one of the largest producers of fox pelts in the world, with an estimated 16,000 farm-bred females, and an average of 90 females per farm [25]. On average, 3.7 and 3.1 pups are born and weaned per farm-bred female, respectively [26]. This makes approximately 50,000 new-born and weaned pups a year which, together with females, are a potential source of escapees from fur farms. In Poland, red fox farming started in 1924 [27], so these activities have been going on for almost 100 years. This means that introgression between wild and farm red foxes may have been taking place during almost a century of farming. Despite the fact that fur farms are obliged to take bio-security measures to prevent animals from escaping, it cannot be ruled out that some individuals do escape from them. However, there are no detailed reports on the numbers of escapees (fur-breeders are unlikely to report the numbers of foxes that have escaped from their farms).

Farm foxes reflect many generations of selective breeding for a number of traits important for fur breeders. This predisposes them to success in human-altered environments [23]. Therefore, gene transfer from farm red foxes to wild individuals via introgression would appear to be quite a real threat. The fact that fox farms are situated across almost the entire country also increases the risk of introgression. This means that introgression can spread from many hybridization spots. However, red fox farms in Poland are not evenly distributed. Most foxes are bred in the east of the country (33.1% of the whole breeding stock) and in the province of Wielkopolska, located in the west-central part of the country (14.8%), and the least in the west (6.1%) [25]. Despite their different habitat preferences, non-native farm foxes do come into contact with native red fox populations, potentially affecting them through genetic admixture and the loss of locally adaptive alleles [21,28].

There have been few studies of genetic introgression between farm red foxes and their wild counterparts. As the main research aim, genetic introgression was investigated in North America by Sacks et al. [28], Lounsberry et al. [5], Akins et al. [29] and Cross et al. [2]. It was also examined and discussed as a secondary aim in research that focused on other objectives [22,23,30]. To the best of our knowledge, genetic introgression in red foxes in Poland has been studied only by Horecka et al. [31], who came to the conclusion that there might be some slight admixture between farm and wild red foxes in south-eastern Poland.

Both farm and wild red foxes belong to the same species; there is no reproductive isolation between them. However, the Eurasian wild red fox belongs to the Holarctic clade, while the farm red fox, of North American ancestry, belongs to the Nearctic clade [21,32]. According to Aubry et al. [32], both clades diverged ca. 400,000 years ago. The two populations thus have a different ancestry. Genetic introgression in this case would not involve two different species, but two populations of the same species that have faced different selection pressures.

The main objective of this study was to investigate the genetic introgression of red foxes bred on fur farms in Poland into the wild native red fox population, and to assess its level and spread within the country. We also evaluated the impact of a geographic barrier (the River Vistula) and isolation by distance on gene flow between two native red fox subpopulations, isolated from each other by the river, and their genetic differentiation.

## 2. Materials and Methods

### 2.1. Sampling Sites

**Figure 1:**
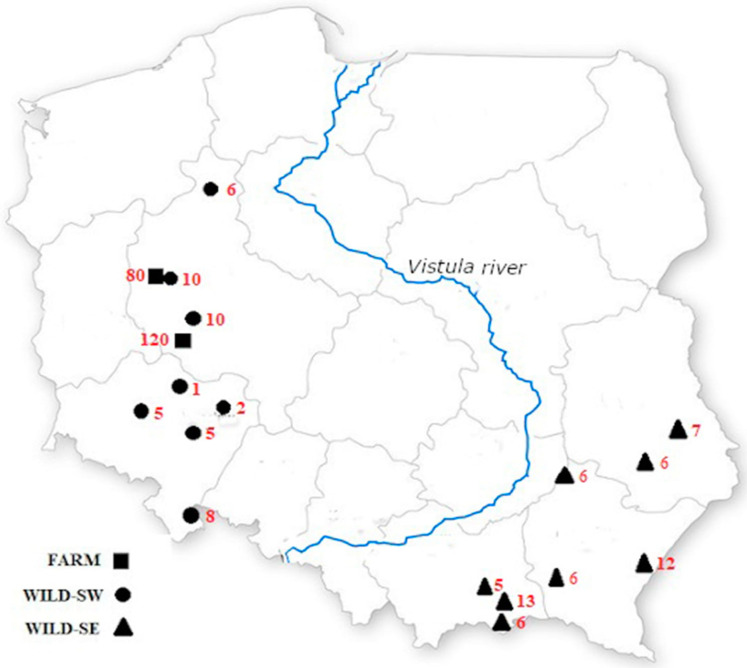
Map of the study area, sampling sites and their location.

The study area covered two regions of Poland, about 500 km apart, and separated by the River Vistula (Wisła). A total of 308 tissue samples (tongue slivers) were used in this study. The samples from unrelated farm foxes (*n* = 200, 85 males and 115 females, denoted FARM) were obtained from two fur farms (*n*_1_ = 120 and *n*_2_ = 80) located in western Poland, whereas those from wild foxes (*n* = 108, 52 males, 51 females, 5 sex unknown) came from 16 locations evenly distributed across the study area (*n*_1_ = 47 individuals, 8 sampling sites in south-western Poland, denoted WILD-SW; *n*_2_ = 61 individuals; and 8 sampling sites in south-eastern Poland, denoted WILD-SE). To ensure the lack of any relationship between the farm animals, their pedigrees, available in farm documentation, were meticulously analysed. The map with sampling sites is shown in Figure 1. We did not need permission from the ethics committee for this experiment, because all the tissue samples were taken post mortem. The samples from the farm foxes were collected after they had been killed at the end of the farm season, while those from wild foxes were collected after they had been killed by hunters or in road accidents.

### 2.2. Microsatellite Amplification and Genotyping

Genomic DNA was extracted according to the protocols used in the ARK Genomics Lab (currently Edinburgh Genomics) [33]. The tissue samples from all the foxes (*n* = 308) were genotyped at 24 autosomal microsatellite loci (AHT137, FH2613, FH2097, FH2980, FH3970, FH3241, FH3713, FH2295, FH3775, FH3824, FH3771, FH3287, FH4001, REN135K06, REN210I14, REN307J23, REN88H03, REN258F18, REN248F14, REN64E19, REN252E18, REN75M10, ZUBECA6 and UOR4101) using previously published primers [34,35,36,37,38,39]. Our earlier study [40] showed that the selected set of canine-derived microsatellite markers robustly amplified red fox DNA. The polymerase chain reaction (PCR) was performed using Qiagen Multiplex PCR and the thermal profile recommended in the manufacturer’s protocols, i.e., 95 °C for 15 min, then 30 cycles of 94 °C for 30 s, 55 °C for 90 s, 72 °C for 60 s; final extension 60 °C for 30 min. The PCR products were electrophoresed along with the Genescan 500 LIZ internal size standard (Applied Biosystems, Foster City, CA, USA) on an ABI 3130XL Genetic Analyzer (Applied Biosystems). Allele sizes were scored using GeneMapper v4.0 (Applied Biosystems). The number and percentage of successfully genotyped individuals are given in Supplementary Table S1. The chromosomal location of the markers in the canine genome (this information is not available for the red fox) can be found in Zatoń-Dobrowolska et al. [40].

### 2.3. mtDNA Amplification and Sequencing

Mitochondrial DNA was extracted using the Sherlock AX kit (A&A Biotechnology, Gdańsk, Poland). Eighty nine samples (48 from farm foxes and 41 from wild foxes) were used to study maternal gene flow from farm to native populations.

Two regions of mtDNA were amplified: a 878-bp segment of the cytochrome b gene and a 443-bp segment of the D-loop. The PCR mixtures were prepared using a DreamTaq Master Mix kit (Thermo Scientific, Waltham, MA, USA) according to the manufacturer’s protocol. A new set of primers for both sequences, designed by the authors of this study, as well as the thermal cycle conditions for both markers were described by Zatoń-Dobrowolska et al. [41]. The mtDNA sequences (the newly detected ones were submitted to GeneBank) used in this study were previously tested by Zatoń-Dobrowolska et al. [41].

The PCR products were purified and sequenced using an ABI BigDye Terminator v3.1 cycle sequencing kit (Applied Biosystems) and ABI 3730 capillary sequencer (Applied Biosystems) for cytochrome b (sequenced from primer VVGluF3) and D-loop (sequenced from primer VVCRR2). The whole lengths of sequences of the two regions combined into concatenated haplotypes were used to investigate mitochondrial lineages.

### 2.4. Statistical Analyses

#### 2.4.1. Microsatellites

Initially, we estimated basic parameters of genetic diversity for both red fox populations (FARM, and wild divided into two groups: WILD-SW, WILD-SE). The observed (H*_O_*) and expected (H*_E_*) heterozygosity, the inbreeding coefficient (F*_IS_*) and the population genetic differentiation (F*_ST_*) were estimated using the *hierfstat* package in R [42]. The number of alleles (NoA) and Hardy–Weinberg equilibrium tests for each locus were performed using the *pegas* package [43]. Allelic richness (A*_R_*) and null allele frequencies were calculated using the *PopGenReport* package [44].

Conventional *F*-statistics were used to estimate gene flow between WILD-SW and WILD-SE [45]. The statistical significance of F*_IS_* and F*_ST_* was verified with the *diveRsity* package [46]. The Mantel test, performed using *mantel.randtest* function [47,48], was applied to investigate the relationships between genetic distance and both the geographic barrier (the River Vistula) and geographic distances. Linearized *F*_ST_ estimates (*F_ST_*/(1 − *F_ST_*), were used as a measure of genetic distance [49]. The matrix of genetic differentiation consisted of the pair-wise *F_ST_*-matrix. The barrier matrix contained 1 if the samples were separated by the River Vistula and 0 if they were not. The matrix of geographic distance contained Euclidean distances (in km) between localities. The Mantel test was applied to study the association (significance of correlation) between the genetic differentiation matrix and the other two matrices. The hierarchical analysis of molecular variance (AMOVA), using the *poppr* package [50,51], was performed to partition variance into components resulting from differences among populations, among localities within populations and among individuals within localities [52,53]. The R software environment [48] was used to run all packages in this part of the statistical analyses.

To evaluate the level of admixture between farm and wild foxes (to detect signals of non-indigenous introgression) the Bayesian clustering approach implemented in Structure v. 2.3.4 [54,55] was used. To assign individuals to genetic clusters (K), correlated allele frequencies with an admixture model were used, varying K from 1 to 10. Twenty replicates for each K with 500,000 burn-ins and 750,000 replicates were used. The Delta K method [56], combined with the standard prediction of K based on plotted mean ln probability of K (L(K)), were applied to analyse the Structure results. Both plots (Delta K and L(K)) were calculated using Structure Harvester [57]. The second approach was the entropy-based method used in the LEA package [58]. The R function *snmf* included in LEA was used to estimate the individual admixture coefficients from the genotypic matrix, and then to provide least-squares estimates of ancestry proportions (this helps to select the number of ancestral populations (K) best explaining the genotypic data.

For all assignments, 95% credibility intervals were estimated to assess confidence in the assignment of individuals. This was done using the *p.function* in R [59]. These analyses were performed on the FARM and wild foxes (WILD-SW and WILD-SE combined).

To identify genetic clusters in the studied red fox populations, principal component analysis (PCA) for microsatellites was performed with the *adegenet* package in R [60]. This approach was used to supplement Bayesian clustering because it does not impose admixture models or assumptions of Hardy–Weinberg equilibrium on the wild and farm red foxes studied. For this analysis the loci with null allele frequencies exceeding 8%, and those for which the 95% confidence interval for null allele frequencies included zero were removed. One allele was removed from the remaining homozygous loci (those that did not exceed the 8% threshold) for which null alleles were detected. The threshold of 8% for null alleles was used because the presence of null alleles within the range of frequency above 5–8% may bias estimates of population differentiation [61].

#### 2.4.2. mtDNA

After reading, editing and chromatogram quality-checking [62], the sequences for mtDNA fragments found in FARM, WILD-SW and WILD-SE were converted to fasta format using Bioedit [62]. The Basic Local Alignment Search Tool (BLAST) was used to search the nucleotide database in GenBank to determine haplotypes for each sample. Then, the sequences were compared with their top BLAST hits in MEGA6 [63]. The haplotypes were finalized when the sequences had no bases mismatching those of the reference haplotypes. After both haplotypes had been assigned to each individual, they were combined into a single concatenated haplotype (hereafter “haplotype”) for each individual. Using these haplotypes, and an infinite site model of DNA sequences and pair-wise deletion of missing data, a haplotype network was constructed with the use of the *pegas* package in R [43]. The nomenclature rules proposed by Statham et al. [64] were applied.

## 3. Results

### 3.1. Genetic Diversity Parameters

The genetic diversity parameters estimated for FARM, WILD-SW and WILD-SE are shown in Table S2 (Supplementary Materials). In FARM we were able to use only 22 of the 24 microsatellite markers because two (FH4001 and FH2295) failed to amplify. The number of alleles per microsatellite locus (NoA) ranged from 1 to 12 (average 5.14) in FARM, from 1 to 43 (average 13.3) in WILD-SW, and from 1 to 42 (average 13.87) in WILD-SE. Three loci (REN25E18, REN248F14, UOR4101) in FARM and one locus in both WILD-SW and WILD-SE (REN248F14) were monomorphic. Allelic richness (A*_R_*) estimated over all loci was 4.22 for FARM, 13.33 for WILD-SW and 13.87 for WILD-SE. Observed heterozygosity (H*_O_*) estimated over all loci was the lowest for FARM (0.47), while WILD-SW and WILD-SE had higher and comparable H*_O_* (0.65 and 0.66, respectively). The same applied to expected heterozygosity (H*_E_*): higher values of H*_E_* over all loci were estimated for WILD-SW and WILD-SE (0.70 and 0.71, respectively), whereas in FARM it was 0.48.

The inbreeding coefficients (F*_IS_*) estimated over all loci were significant (*p* < 0.05) for all three red fox populations studied (Supplementary Materials, Table S2). However, the evidently highest value of F*_IS_* was estimated for FARM (0.18), whereas WILD-SW and WILD-SE had lower and comparable values of F*_IS_* (0.08 and 0.07, respectively). The F*_IS_* values estimated per locus were significant (*p* < 0.05) for two loci in FARM, four loci in WILD-SW and six loci in WILD-SE, indicating deviations from Hardy–Weinberg equilibrium.

The genetic diversity parameters (NoA, A*_R_*, H*_0_*, H*_E_*) indicate much higher genetic diversity among the wild foxes (both WILD-SW and WILD-SE) in comparison to the farm foxes.

AMOVAs performed for FARM, WILD-SW and WILD-SE together showed that 31.50% of the total genetic variance was caused by variability between populations, 2.60% was caused by variability between localities within populations, whereas 65.90% was caused by variability within localities. Partitioning of the total genetic variance into components performed for native populations only (WILD-SW and WILD-SE combined) revealed that a very small part of the total variance (0.45%) was caused by variability between populations, 6.98% was caused by variability between localities within populations, while as much as 92.57% was caused by variability within localities.

### 3.2. Genetic Differentiation and Genetic Structure

**Figure 2:**
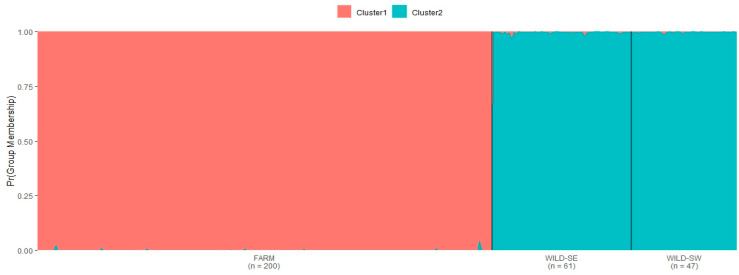
Bar plot showing probability of assignment for individual wild and farm red foxes at the most highly supported value of genetic clusters (K = 2).

**Figure 3:**
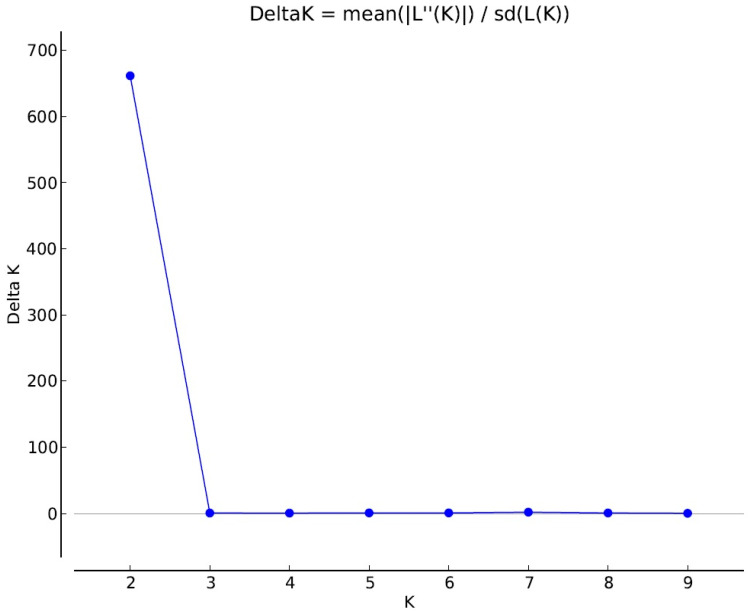
The Delta K plot showing the highest pick at K = 2 (the most probable number of genetic clusters).

The genetic structure analysis indicated two genetic clusters as being the most probable number of genetically distinct populations (Figure 2). The Delta K plot (Figure 3) showed the highest peak at K = 2, supporting the division into two genetic clusters with no or a very weak signals of admixture. The L(K) plot also indicated a break in linearity and the lowest standard deviation for K = 2 (plot not shown). For K = 2, the WILD-SW population clustered with the WILD-SE population, while the second cluster corresponded to the FARM population (Figure 2). Two additional analyses using the Structure package performed to increase the sensitivity of detection of a small level of admixture between farm and wild foxes (for K = 3 and K = 4), confirmed our earlier findings (farm and wild foxes are two separate clusters), and detected a more complex genetic structure of farm foxes (Supplementary Figures S1 and S2). The second analysis of the genetic structure (using the LEA package), performed to validate the Structure results, also indicated a clear minimum at K = 2, suggesting two genetic clusters (Supplementary Figures S3 and S4).

The 95% credibility intervals of farm foxes overlapped the 100% assignment to the FARM genetic cluster in all individuals. The same was observed for wild foxes—all 95% credibility intervals overlapped the 100% assignment to the WILD (WILD-SW + WILD-SE) genetic cluster in all cases. This indicates with high probability that there is no admixture between farm and wild red foxes.

**Figure 4:**
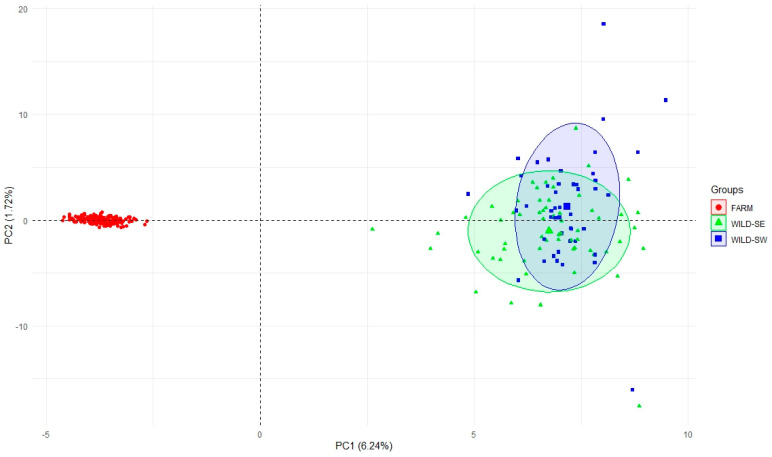
Cluster composition and population differentiation of the studied red foxes.

PCA carried out to show relationships between multi-locus genotypes in two-dimensional space (Figure 4) revealed that principal components (PCs) with eigenvalues > 1.0 explained 14.02% of the total variation in the data. Together, PC1 and PC2 accounted for 7.96% of the total variance (6.24% and 1.72%, respectively). The first two components were thus retained for further analysis. The scatter plot (Figure 4) showed that PC1 completely separated FARM and native foxes (WILD-SW and WILD-SE).

The fixation index (F*_ST_*) estimated over all loci showed a significant genetic differentiation between FARM and WILD-SW (F*_ST_* = 0.31, *p* < 0.05), and FARM and WILD-SE (F*_ST_* = 0.30, *p* < 0.05), but a non-significant genetic differentiation between WILD-SW and WILD-SE (F*_ST_* = 0.004).

The Vistula (the largest river in Poland), separating WILD-SW and WILD-SE, did not significantly limit gene flow between these populations. The standard Mantel test done for microsatellite markers indicates a non-significant correlation between genetic differentiation and the presence of this geographic barrier (r = 0.48, *p*-value = 0.69). Isolation by distance of both subpopulations also had no significant effect on their genetic differentiation (r = −0.44, *p*-value = 0.65).

### 3.3. mtDNA Concatenated Haplotype Frequencies and Haplotype Network

**Figure 5:**
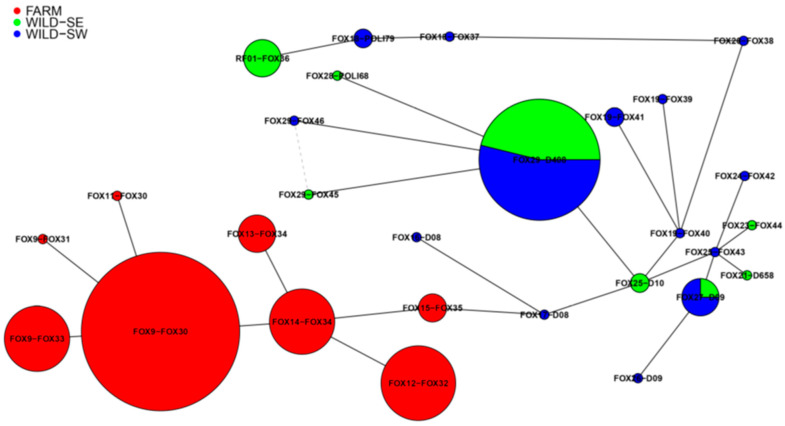
Haplotype network showing relationships between concatenated haplotypes of farm and wild red foxes. The size of the circle represents the haplotype frequency.

Mitochondrial DNA haplotype frequencies (the 878-bp segment of the cytochrome b gene and the 443-bp segment of the D-loop) found in FARM, WILD-SW and WILD-SE, as well as the haplotype network indicating relationships between haplotypes of FARM, WILD-SW and WILD-SE, are shown in Table 1 and Figure 5. In total, we found 28 different haplotypes: 8 in FARM and 20 in native foxes (WILD-SW+WILD-SE). None of the haplotypes detected in FARM was found in native foxes. Of the 20 different haplotypes observed in the native foxes, 14 were found in WILD-SW and 8 in WILD-SE. Only 2 different haplotypes (FOX29-D408 and FOX27-D09) were common to both native populations.

The most numerous haplotype in FARM was FOX9-FOX30 (*n* = 17), followed by FOX12-FOX32 (*n* = 8), and FOX14-FOX34 and FOX9-FOX33 (for both *n* = 7). In WILD-SW and WILD-SE the most numerous haplotype was FOX29-D408 (*n* = 7 and *n* = 6, respectively), followed by RF01-FOX36 (*n* = 4, WILD-SE) and FOX27-D09 (*n* = 3, WILD-SW).

The haplotype network (Figure 5) shows two genetically distinct clusters: FARM, which is completely separated from native red foxes, and WILD-SW and WILD-SE, which are clustered together and have two haplotypes in common.

## 4. Discussion

The genetic introgression of farm-bred individuals into native populations may put the long-term viability of local populations at risk [5]. Although introgressive hybridization and admixture may have beneficial results through the introduction of new alleles (especially into small, fragmented and isolated populations) [65], the local populations viability could be threatened by the spread of infectious diseases and competition with hybrids for environmental resources [4]. Therefore, it is important to understand in detail how the accidental introduction of farm-bred individuals into wild populations may change the genetic fitness and viability of wild populations. A detailed understanding of genetic introgression between wild individuals and escapees from farms is critical to the management of species and may provide insight into evolutionary processes [66,67,68].

In the present study we compared samples of DNA (both nuclear and mtDNA) taken from three groups of red foxes: farm foxes, wild foxes living in the area where these farms were located, and wild foxes from an area several hundred km distant from the first two and isolated by a geographic barrier (the River Vistula). We assumed that if the flow of non-native alleles from fox farms into the wild population of the red fox was not prevented, admixture analysis should show successively higher levels of introgression with increasing proximity to the locations of fur farms. Otherwise, admixed red foxes should not be observed at all or only in the immediate vicinity of the farms.

The results of our research are consistent and indicate no or very limited genetic introgression between farm and native red foxes in Poland. Both the analysis of the genetic structure based on microsatellite markers (using the Structure package and PCA) as well as the comparison of haplotypes between FARM, WILD-SW and WILD-SE (haplotype network) indicate the coexistence of two genetic clusters (wild and farm red foxes) with no or very little admixture of non-native alleles. Additional support for this observation is provided by: (1) genetic diversity parameters estimated for the studied red fox populations. Even if they are not measures of genetic differentiation, they may suggest a genetic distinctiveness between FARM (NoA, A*_R_*, H*_0_*, H*_E_* are much lower than in native individuals), and WILD-SW and WILD-SE (the genetic diversity parameters are very similar in both groups, indicating that this is actually one population). Indeed, the cross-entropy criterion estimated when analysing the genetic structure of wild foxes (using the LEA package) as well as PCA performed for WILD-SW and WILD-SE combined, clearly indicated one genetic cluster (Supplementary Materials, Figures S5 and S6); (2) significant genetic differentiation (F*_ST_*) between FARM and both WILD-SW and WILD-SE; and (3) no genetic differentiation between WILD-SW and WILD-SE. Furthermore, the Mantel test showed that the Vistula, the longest river in Poland and the ninth-longest river in Europe (1047 km in length, 1.5 km at its widest point, the riverbed not artificially reconstructed), with sandy islands in the course of the river, was not a geographic barrier effectively preventing gene flow between WILD-SW and WILD-SE (this is probably one of the reasons for the genetic homogeneity of native red foxes). Therefore, it can be assumed that the Vistula would not be an effective barrier to the flow of non-native alleles from WILD-SW (if they were admixed with FARM) or farm escapees to WILD-SE. Neither was any signal of isolation by distance found, which confirms the unrestricted gene flow between WILD-SW and WILD-SE.

Despite the fact that our study does not indicate that the River Vistula is an important geographic barrier to gene flow between the studied subpopulations of the red fox, some authors [69,70] suggest that larger rivers may limit the dispersal (gene flow) of foxes. Wandeler et al. [70] reported that the River Limmat influenced red fox colonization in the city of Zürich (Switzerland). Population differentiation was consistently higher between foxes on opposite sides of the river than those on the same side. Indirect evidence to support the role of rivers in limiting gene flow between red fox populations in Poland was provided by Bourhy et al. [69]. They studied the two main phylogenetic groups of the rabies virus in Poland (found in foxes and raccoon dogs, *Nyctereutes procyonoides*): the Central European cluster and the North Eastern European cluster. These two groups were found to be separated by the River Vistula, indicating limited movements of the host species (e.g., the red fox) across the river. According to Stojak and Tarnowska [71], however, the contemporary genetic structure of small mammals in Poland indicates that the Vistula could not be an important barrier to gene flow. It seems that the factors enabling red foxes to cross the Vistula are the low water levels often recorded in summer (in some stretches it can be as shallow as ca 40 cm) and the numerous sandy islands in the riverbed. In addition, its banks are connected by 57 bridges and during the winter the river sometimes freezes over.

Genetic introgression is more often observed when the ranges of closely related species overlap [72], or one species is rare and individuals have to find mates from a closely related species [73]. In the second situation (when one species exists at low density) asymmetric introgression may take place [74]. Asymmetric gene flow may also be caused by sex-biased dispersal or philopatry. The male-biased dispersal leads to differentiated mtDNA, while nuclear DNA remains homogeneous. In contrast, if female-biased dispersal takes place, mtDNA remains homogeneous, but nuclear DNA exhibits differentiation [75].

In red foxes, males and females have different dispersal dynamics. Males migrate at higher rates and for longer distances, whereas females are philopatric [76]. Therefore, greater nuclear than mitochondrial DNA transfer should be expected. In our study, we did not use Y-chromosome markers to study male-mediated gene flow. However, because we did not detect the genetic mixing of either nuclear or mitochondrial DNA but did find two well-defined and separated genetic clusters (wild and farm foxes), testing whether gene flow (if detected) was male-biased was rather less important for our study.

The geography of admixture between native and non-native individuals is also an important issue when investigating introgression. In the situation where fox farms are scattered throughout the country (as in our research), introgression is not limited to distribution margins, as in the wave model, but is rather caused and accelerated by hybridization spots within the range of a native species [17]. This should be expected in Poland in the context of introgression between native and farm red foxes (their ranges overlap). However, we were unable to test this for lack of any admixture between the examined populations and, as a result, because no admixed individuals could be located.

American researchers [30] studied the effect of distance, landscape type and elevation on maternal (mtDNA haplotypes) and nuclear (microsatellites) gene flow between native and non-native red fox (*V. v. macroura*) populations in Colorado. They reported that native haplotypes were predominant in mountainous areas, while non-native haplotypes were more common in more urbanized and lowland areas. They also confirmed higher male-mediated gene flow. Poland, compared to other European countries and the USA, is a relatively poorly urbanized. Most of its territory is rural or non-urbanized. Additionally, Poland is a rather lowland country with mountain ranges located in the south of the country. Fox farms are located in agricultural and poorly urbanized areas, usually in close proximity to forests and open areas, far from cities and large human clusters. Therefore, we believe that these landscape conditions do not play a significant role in limiting potential genetic introgression between farm and wild red foxes (e.g., spread of farmed fox haplotypes in the wild fox population).

In our previous research [41], we identified 23 concatenated haplotypes in wild foxes and 8 concatenated haplotypes in farm foxes (which were reused in the present study). In this study, of the 23 concatenated haplotypes previously reported, we detected 20 (the missing haplotypes were: 3-P02, FOX22-FOX44 and KHARKIV10-RS56). The distribution of these haplotypes between WILD-SW and WILD-SE was not similar (only two haplotypes were common to WILD-SW and WILD-SE). It turned out that, in contrast to microsatellite markers, the maternal gene flow between WILD-SW and WILD-SE was limited. We believe this may be attributed to the females’ philopatry [76]. As we mentioned earlier, female red foxes occupy home ranges or territories while the males are migratory. We do not believe that the River Vistula was a barrier to the effective flow of maternal genes (it was shown by the Mantel test and suggested by Stojak and Tarnowska [71]).

Despite the fact that we did not detect strong signals of admixture between farm and wild foxes, Horecka et al. [31], who investigated the origin of farm foxes bred in Poland, reported that some degree of genetic introgression between Polish farm and wild populations was possible. These authors carried out their study using samples collected in south-eastern Poland. The mtDNA analyses were conducted using concatenated nucleotide sequences of MT-CO1 (mitochondrially encoded cytochrome c oxidase I) and MT-ATP6 (mitochondrially encoded ATP synthase 6) genes. They found a haplotype common to wild Polish foxes and wild North American animals (ancestors of the farm foxes). This led them to conclude that wild foxes with an admixture of farm fox alleles could occur in Poland. The low introgression rates reported by Horecka et al. [31] and the results of our study indicate that this process should be considered a rather uncommon event and would contribute little to the genetic structure of the native red fox population. That is why the farm and wild foxes under study remain two well-defined genetic clusters.

Signals of admixture between farm and native red foxes on the island of Newfoundland (Canada), where a large fox farm has been operating for more than 30 years, were evaluated by Lounsberry et al. [5]. They compared mtDNA sequences and nuclear microsatellite genotypes (21 loci) of 93 individuals from the fox farm to those of 79 contemporary wild foxes sampled from across the island. For reference, they used 12 historical museum specimens of wild eastern Canadian red foxes. Those authors reported that many mtDNA haplotypes were shared among compared groups of foxes (farmed, contemporary wild and historical wild). This was expected, given the eastern Canadian origin of fur-farming. There was significant differentiation (F*_ST_* = 0.14, *p* < 0.001) between farm and wild foxes, indicating little gene flow between the two groups of foxes. Admixture and principal component analyses supported the clear separation of farm and wild red foxes. The authors concluded that gene flow from the Newfoundland red fox farm to the native population had caused little, if any, admixture. However, they suggested that the apparent admixture could have reflected a type I error, which was expected to occur in the sample on the basis of chance alone.

One of the possible reasons for the very limited non-native introgression from farm to wild foxes could be the monogamous mating system of red foxes. This was suggested by Sacks et al. [28]), who studied the extent of hybridization between an endemic subspecies of red fox (*V. v. patwin*) and introduced, phylogenetically divergent red foxes resulting from a century of domestication. They found that hybridization was primarily restricted to a narrow zone where the two populations came into contact. The authors speculated that native foxes rejected introduced individuals. They also hypothesized that the monogamous mating system of red foxes could enhance their resistance to hybridization. According to Rheindt and Edwards [17], a rare non-native genotype would find it hard to introgress beyond a hybrid zone (e.g., within the range of a native species), because genetic drift would counteract its spread. One might hypothesize that many years of different selection pressures in farm and wild foxes (selective breeding vs. natural selection) and their different origins (North American vs. Eurasian) have led to significant genetic differentiation between both red fox populations, which could consequently cause a reproductive barrier (or at least limited mating).

## 5. Conclusions

Our research indicates that the genetic integrity of the native red fox population in Poland is currently not threatened by an inflow of non-native alleles from farm foxes. We found that the native red fox population, though divided by the River Vistula, in fact comprise one single genetic cluster without or very little admixture of non-native alleles. Given the relatively large scale of our study, we may be justified in stating that it does appear to reflect the real picture of genetic introgression between farm and wild red foxes in the country. Nevertheless, even though we did not detect strong signals of admixture between farm and native red foxes, the genetic structure of the Polish red fox population needs to be monitored so that relevant protective measures can be implemented as needed. This will allow wildlife organizations and fur breeders (1) to develop a model for effectively controlling the inflow of non-native alleles to the Polish red fox population, and (2) to avoid a scenario when the cause of, and then the solution to the problem is sought only when the species is already at risk.

## Figures and Tables

**Table 1:** Cytochrome b and D-loop concatenated haplotype frequencies in the studied red foxes.

Concatenated Haplotype	FARM ^a^	WILD-SW	WILD-SE
FOX9-FOX30	17		
FOX15-FOX35	3		
FOX13-FOX34	4		
FOX14-FOX34	7		
FOX9-FOX33	7		
FOX12-FOX32	8		
FOX9-FOX31	1		
FOX11-FOX30	1		
FOX18-FOX37		1	
FOX29-D408		7	6
FOX29-FOX45		-	1
FOX27-D09		3	1
FOX28-POLI68		-	1
FOX25-D10		-	2
FOX19-FOX41		2	-
FOX19-FOX39		1	-
FOX16-D08		1	-
FOX21-D658		-	1
RF01-FOX36		-	4
FOX17-D08		1	-
FOX18-POLI79		2	-
FOX29-FOX46		1	-
FOX20-FOX38		1	-
FOX25-FOX43		1	-
FOX19-FOX40		1	-
FOX23-FOX44		-	1
FOX26-D09		1	-
FOX24-FOX42		1	-

^a^ taken from our previous study [41].

## Data Availability

The data presented in this study (genotypes of the studied individuals) are openly available in Dryad at https://doi.org/10.5061/dryad.np5hqbzsq.

## Supplementary Materials

The following are available online at https://www.mdpi.com/article/10.3390/genes12050637/s1, Figure S1: Bar plot produced for K = 3 inferred by the Structure package [54,55], Figure S2: Bar plot produced for K = 4 inferred by the Structure package [54,55], Figure S3: Bar plot showing probability of assignment for individual farm (FARM, green colour) and wild (WILS-SW+WILD-SE, red colour) red foxes at the most highly supported value of genetic clusters (K = 2), inferred by the LEA package [58], Figure S4: The cross-entropy criterion used in the LEA package [58] at the most highly supported value of putative genetic clusters, K = 2. The cross-entropy criterion is a value based on the prediction of masked genotypes to evaluate the error of ancestry estimation. The cross-entropy criterion helps to choose the best number of ancestral population (K). A smaller value of cross-entropy means a better run in terms of prediction capacity, Figure S5: The cross-entropy criterion used in the LEA package [58] at the most highly supported value of putative genetic clusters, K = 1. The cross-entropy criterion is a value based on the prediction of masked genotypes to evaluate the error of ancestry estimation. The cross-entropy criterion helps to choose the best number of ancestral population (K). A smaller value of cross-entropy means a better run in terms of prediction capacity, Figure S6: Principal component analysis (PCA) showing the genetic differentiation of WILD-SW and WILD-SE in Poland. The first two components (PC1 and PC2) accounting for 4.19% of the total variance (2.15% and 2.04%, respectively) were retained for the analysis. The scatter plot of the first two principal components displayed in a two-dimensional space shows no genetic separation between WILD-SW and WILD-SE (both sub-populations form one genetic cluster). The analysis was performed with the adegenet package in R [60], Table S1: The number and percentage of genotyped individuals, Table S2: Parameters of genetic diversity estimated for the studied farm and wild red foxes.
