# Supplementary material for: Insight into the Genetic Population Structure of Wild Red Foxes in Poland Reveals Low Risk of Genetic Introgression from Escaped Farm Red Foxes
Source: Genes (Basel). 2021 Apr 25;12(5):637. doi: 10.3390/genes12050637 (PMC8146073; doi:10.3390/genes12050637)
Supplement: Supplementary file 1 [file genes-12-00637-s001.zip › genes-1144392-supplementary.pdf]

Table S1. The number and percentage of genotyped individuals.

Locus	Farm foxes (n=200)		Wild foxes SE (n=61)		Wild foxes SW (n=47)	
	Number of genotyped individuals	% of successfully genotyped	Number of genotyped individuals	% of successfully genotyped	Number of genotyped individuals	% of successfully genotyped
AHT137	199	99.50	61	100	47	100
FH2097	199	99.50	45	73.77	42	89.36
FH2613	200	100	61	100	47	100
FH2980	199	99.50	60	98.36	46	97.87
REN135K06	199	99.50	58	95.08	47	100
REN248F14	199	99.50	60	98.36	47	100
REN258F18	199	99.50	58	95.08	47	100
REN64E19	179	89.50	61	100	47	100
REN88H03	200	100	58	95.08	47	100
UOR4101	199	99.50	59	96.72	47	100
ZUBECA6	200	100	60	98.36	46	97.87
FH3241	194	97.00	59	96.72	47	100
FH3287	198	99.00	53	86.88	40	85.11
FH3713	199	99.50	60	98.36	47	100
FH3771	169	84.50	55	90.16	42	89.36
FH3775	188	94.00	60	98.36	46	97.87
FH3824	200	100	61	100	47	100
FH3970	163	81.50	40	65.57	28	59.57
FH4001	0	0	59	96.72	46	97.87
REN210I14	198	99.00	36	59.02	29	61.70
REN25E18	199	99.50	61	100	47	100
REN307J23	197	98.50	56	91.8	46	97.87
REN75M10	192	96.00	51	83.61	44	93.62
FH2295	0	0	43	70.49	39	82.98

Table S2. Parameters of genetic diversity estimated for the studied farm and wild red foxes.

LOCUS	NoA			A_R			H_O			H_E			F_{IS}		
	FARM	WILD SW	WILD SE	FARM	WILD SW	WILD SE	FARM	WILD SW	WILD SE	FARM	WILD SW	WILD SE	FARM	WILD SW	WILD SE
AHT137	3	9	11	3.00	8.02	8.59	0.67	0.77	0.72	0.60	0.80	0.78	-0.12*	0.04	0.08
FH2097	9	13	16	8.35	12.27	13.87	0.71	0.83	0.73	0.68	0.90	0.90	-0.05	0.08	0.18*
FH2613	8	10	11	4.72	8.51	9.14	0.24	0.45	0.67	0.26	0.55	0.69	0.08	0.18	0.02
FH2980	7	18	20	6.00	16.66	17.38	0.72	0.89	0.85	0.76	0.93	0.94	0.04	0.04	0.09
FH3241	4	12	11	3.98	10.56	9.26	0.49	0.81	0.81	0.51	0.81	0.80	0.03	0.00	-0.01
FH3287	2	5	5	2.00	4.97	4.78	0.38	0.75	0.66	0.38	0.66	0.67	0.00	-0.13	0.02
FH3713	5	28	27	3.73	21.47	19.41	0.61	0.89	0.93	0.60	0.92	0.91	-0.02	0.03	-0.02
FH3771	10	21	23	6.85	18.70	18.99	0.69	0.83	0.96	0.69	0.93	0.93	0.00	0.11	-0.03
FH3775	3	4	3	3.00	3.60	2.72	0.44	0.61	0.38	0.42	0.51	0.49	-0.05	-0.19	0.21
FH3824	10	36	36	7.42	29.11	26.50	0.77	0.98	0.85	0.76	0.97	0.96	-0.01	-0.01	0.12*
FH3970	6	13	14	5.45	13.00	13.02	0.66	0.68	0.83	0.68	0.89	0.88	0.03	0.24*	0.07
FH4001	nPCR[a]	43	41	nPCR	33.00	28.30	nPCR	0.89	0.92	nPCR	0.98	0.96	nPCR	0.09	0.04
FH2295	nPCR	4	5	nPCR	3.64	4.61	nPCR	0.31	0.47	nPCR	0.33	0.44	nPCR	0.06	-0.06
REN210I14	4	7	6	3.44	6.97	5.95	0.19	0.72	0.81	0.40	0.76	0.74	0.53*	0.05	-0.09
REN25E18	1	2	2	1.00	2.00	2.00	0.00	0.21	0.31	0.00	0.23	0.26	-	0.06	-0.18*
REN307J23	3	12	12	3.00	11.32	10.37	0.39	0.74	0.68	0.42	0.85	0.85	0.08	0.13	0.21*

REN75M10	9	10	12	5.43	8.91	10.87	0.70	0.66	0.76	0.74	0.86	0.88	0.05	0.23*	0.13
REN135K06	5	7	7	4.83	6.46	6.71	0.69	0.66	0.69	0.67	0.66	0.74	-0.02	0.00	0.07
REN248F14	1	1	1	1.00	1.00	1.00	0.00	0.00	0.00	0.00	0.00	0.00	-	-	-
REN258F18	5	14	14	4.75	12.86	12.92	0.71	0.81	0.69	0.71	0.87	0.86	-0.02	0.07	0.20*
REN64E19	3	7	9	2.96	6.92	8.14	0.23	0.64	0.80	0.25	0.80	0.83	0.05	0.20*	0.03
REN88H03	2	2	3	2.00	2.00	2.98	0.38	0.30	0.19	0.44	0.31	0.24	0.14	0.05	0.20
UOR4101	1	3	2	1.00	2.83	2.00	0.00	0.32	0.17	0.00	0.35	0.21	-	0.08	0.20
ZUBECA6	12	39	42	8.84	31.85	30.73	0.69	0.83	0.85	0.70	0.98	0.98	0.01	0.16*	0.13*
OVERALL	5.14	13.33	13.87	4.22	11.53	11.26	0.47	0.65	0.66	0.48	0.70	0.71	0.18*	0.08*	0.07*

NoA – number of alleles, A_R – allelic richness, H_O – observed heterozygosity, H_E – expected heterozygosity, F_{IS} – inbreeding coefficient, FARM – farm foxes, WILD SW – wild foxes from south-western Poland, WILD SE – wild foxes from south-eastern Poland

* Statistically significant at $P < 0.05$

[a] no PCR product

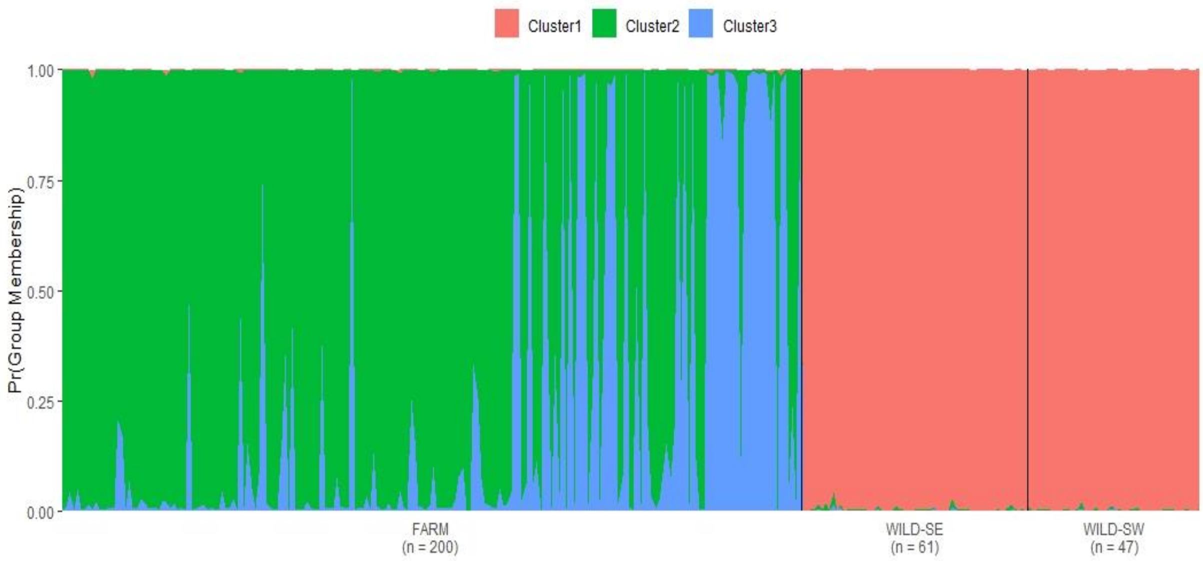

Figure S1. Bar plot produced for K = 3 inferred by the Structure package [54,55].

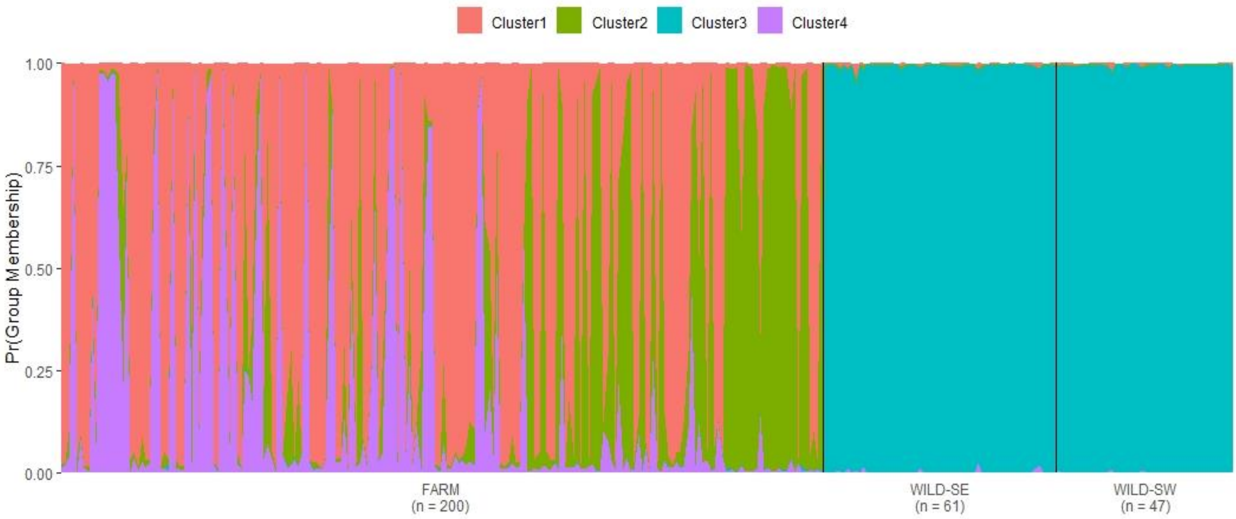

Figure S2. Bar plot produced for K = 4 inferred by the Structure package [54,55].

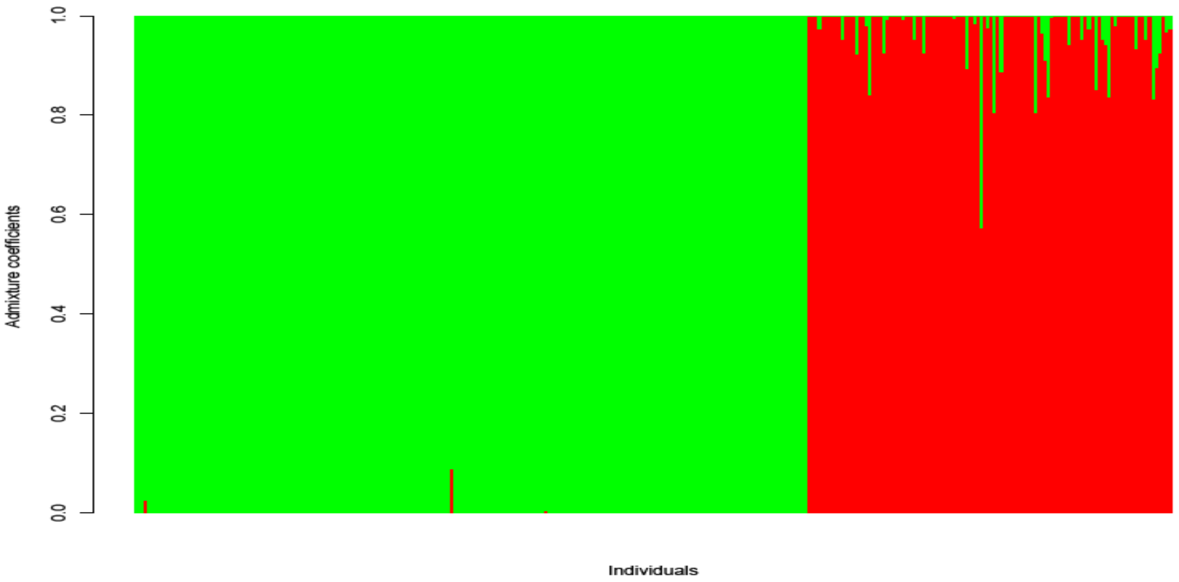

Figure S3. Bar plot showing probability of assignment for individual farm (FARM, green colour) and wild (WILS-SW+WILD-SE, red colour) red foxes at the most highly supported value of genetic clusters (K = 2), inferred by the LEA package [58].

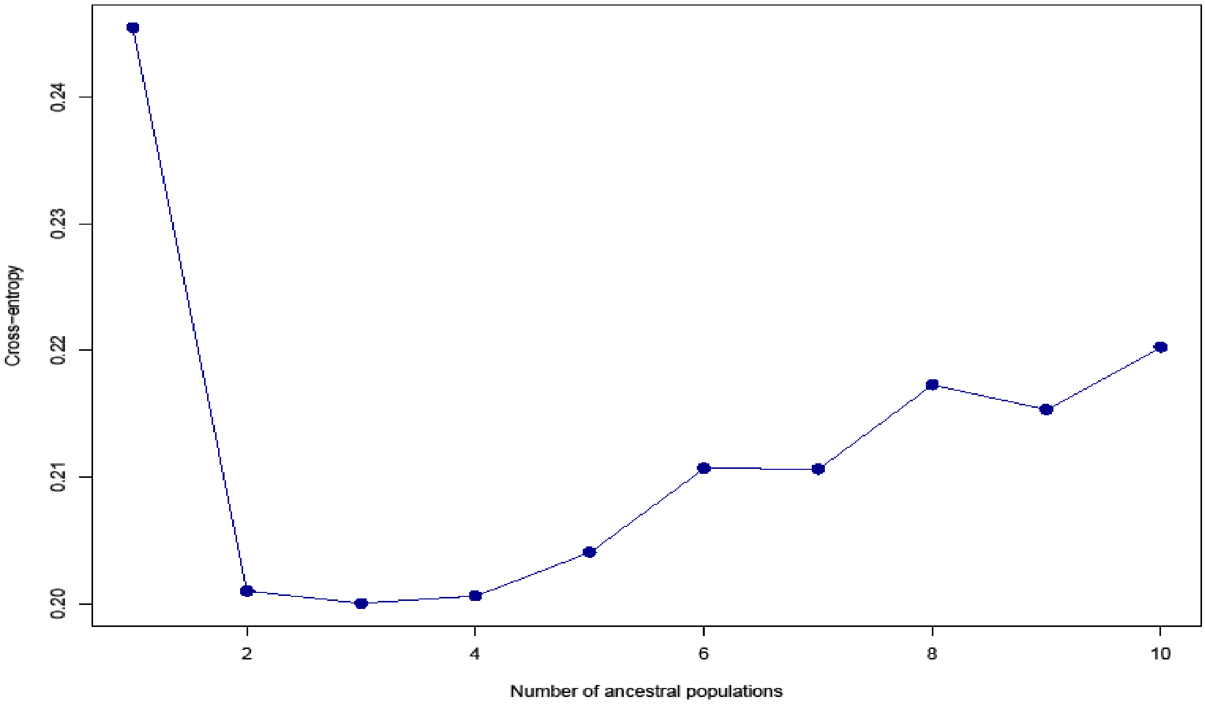

Figure S4. The cross-entropy criterion used in the LEA package [58] at the most highly supported value of putative genetic clusters, K = 2. The cross-entropy criterion is a value based on the prediction of masked genotypes to evaluate the error of ancestry estimation. The cross-entropy criterion helps to choose the best number of ancestral population (K). A smaller value of cross-entropy means a better run in terms of prediction capacity.

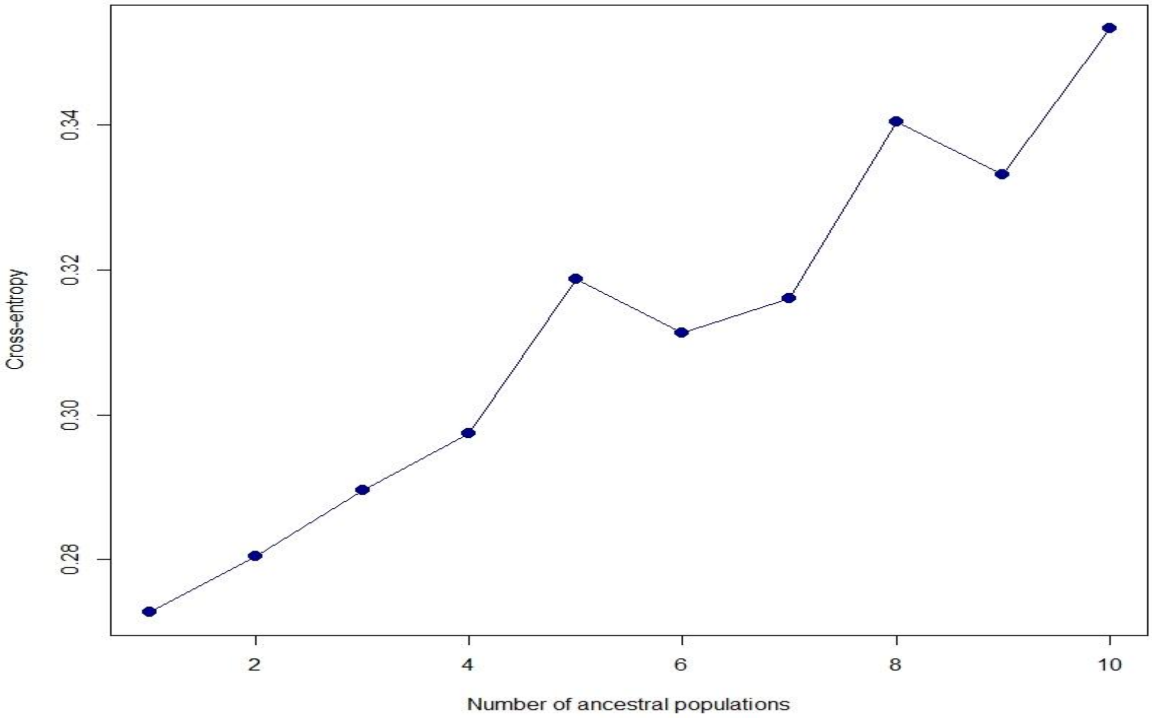

Figure S5. The cross-entropy criterion used in the LEA package [58] at the most highly supported value of putative genetic clusters, K = 1. The cross-entropy criterion is a value based on the prediction of masked genotypes to evaluate the error of ancestry estimation. The cross-entropy criterion helps to choose the best number of ancestral population (K). A smaller value of cross-entropy means a better run in terms of prediction capacity.

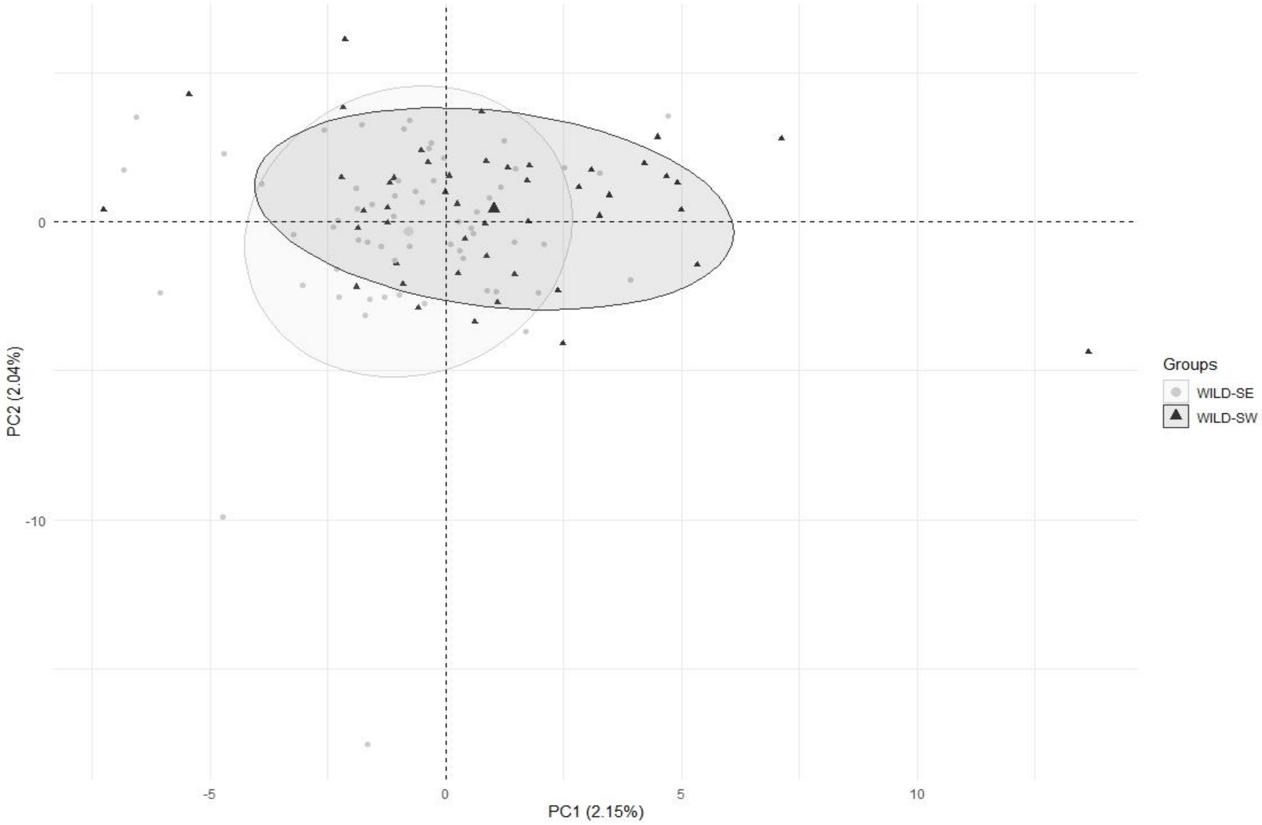

Figure S6. Principal component analysis (PCA) showing the genetic differentiation of WILD-SW and WILD-SE in Poland. The first two components (PC1 and PC2) accounting for 4.19% of the total variance (2.15% and 2.04%, respectively) were retained for the analysis. The scatter plot of the first two principal components displayed in a two-dimensional space shows no genetic separation between WILD-SW and WILD-SE (both sub-populations form one genetic cluster). The analysis was performed with the *adegenet* package in R [60].
